# Dynamics Behaviors of Droplet on Hydrophobic Surfaces Driven by Electric Field

**DOI:** 10.3390/mi10110778

**Published:** 2019-11-14

**Authors:** Jie Liu, Sheng Liu

**Affiliations:** 1 The Laboratory of Transients in Hydraulic Machinery, School of Power and Mechanical Engineering, Wuhan University, Wuhan 430072, China; lll202087@whu.edu.cn; 2 Electronic Manufacturing and Packaging Laboratory, the Institute of Technological Sciences, Wuhan University, Wuhan 430072, China

**Keywords:** dielectrophoresis, droplet, electrohydrodynamics, phase field method, non-uniform electric field

## Abstract

Droplet microfluidic technology achieves precise manipulation of droplet behaviors by designing and controlling the flow and interaction of various incompatible fluids. The electric field provides a non-contact, pollution-free, designable and promising method for droplet microfluidics. Since the droplet behaviors in many industrial and biological applications occur on the contact surface and the properties of droplets and the surrounding environment are not consistent, it is essential to understand fundamentally the sessile droplet motion and deformation under various conditions. This paper reports a technique using the pin-plate electrode to generate non-uniform dielectrophoresis (DEP) force to control sessile droplets on hydrophobic surfaces. The electrohydrodynamics phenomena of the droplet motion and deformation are simulated using the phase-field method. It is found that the droplet moves along the substrate surface to the direction of higher electric field strength, and is accompanied with a certain offset displacement. In addition, the effect of pin electric potentials, surface contact angles and droplet volumes on the droplet motion and deformation are also studied and compared. The results show that higher potentials, more hydrophobic surfaces and larger droplet volumes exhibit greater droplet horizontal displacement and offset displacement. But for the droplet vertical displacement, it is found that during the first revert process, the release of the surface tension can make the droplet with low potentials, small contact angles or small droplet volumes span from negative to positive. These results will be helpful for future operations encountered in sessile droplets under non-uniform electric fields towards the droplet microfluidics applications.

## 1. Introduction

Recently the dynamic behavior of sessile droplets on substrate surfaces have received considerable attention, especially for superhydrophobic surfaces with low adhesion resistance and ultra-low surface energy [1]. The research results of dynamic behaviors of droplets have been widely used in broad applications, such as self-cleaning [2,3], anti-icing [4], heat transfer [5,6], electronics [7,8,9], and microelectromechanical systems [10], etc. Typically, there are two types of ways to drive the dynamic behavior of sessile droplets. One is to make full use of the droplets’ own gravity or surface tension. The gravity can play a major role when the droplet volume is large enough. The dynamic behavior of droplets can be enhanced by increasing tilt angles of substrate surfaces or using a more hydrophobic substrate structure and so on. On the other hand, surface tension plays a major role when the droplet volumes are small enough to ignore their own gravity, which are mainly manifested in promoting the droplet coalescence-driven jump [11,12]. Another method is to apply an external force to drive the dynamic behavior of sessile droplets. The existing methods include applied electric fields [13,14,15,16,17], magnetic fields [18], pressure [19], air flow [20,21,22], laser [23] and so on. In these methods, the electric field driving the dynamic behavior of sessile droplets has been developed as a relatively technology exhibiting promising perspective for the droplet control [24,25]. Takeda et al. [13] experimentally studied the effects of direct current (DC) and alternating current (AC) electric fields on water droplets on superhydrophobic surfaces, demonstrating that superhydrophobic surfaces are beneficial for controlling water droplets through small electric fields. Sakai et al. [14] developed a particle image velocimetry (PIV) system to evaluate the internal fluidity of water droplets moving on a superhydrophobic surface by electric fields. Zhu Y et al. [15] experimentally studied the water droplet behaviors on superhydrophobic surfaces under an increasing AC voltage, and numerically simulated the electrification characteristics of water droplets deposited on hydrophobic surfaces and their influence on a driven discharge in an AC electric field. Wei et al. [16] experimentally conducted the rolling behavior of the water droplet on superhydrophobic surfaces under electrical fields, and built a finite element modeling (FEM) simulation model to indicate that an electrostatic force produced by electrical fields drove a water droplet to roll. Adamiak [17] numerically simulated the deformation of an ideally conducting liquid droplet deposited on the flat dielectric surfaces by solving the capillary Laplace–Young equation. Liu [26] conducted numerical simulations and experiments on the dynamic mechanism of water droplet formation with different applied voltages and droplet distribution, and drove surface discharges on the insulator surface under an AC electric field. However, existing research on electric fields driven dynamic behaviors of sessile droplets is mainly experimental, and the simulation research efforts are few and mainly focused on the polarization analysis of stationary droplets [2,5,27]. In addition, since sessile droplets constitute the three-phase contact line, the simulation research on the dynamic behavior of sessile droplets is naturally complicated [28,29]. The polarization effect during the electric-driven motion and deformation of sessile droplets remains unclear. There is still a need for a robust model of an electric-driven droplet to predict changes in droplet dynamic behaviors. 

In this study, therefore, a phase-filed two-phase flow model under simplified non-uniform electric fields is developed to simulate the electrohydrodynamic behaviors of sessile droplets on hydrophobic surfaces. With this model, the electro-driven motion and deformation of water droplets in the oil phase are analyzed, and the horizontal displacement, vertical displacement and offset displacement of droplets are quantified to express the effect of non-uniform electric fields. In addition, the effects of pin electric potentials, surface contact angles and droplet volumes on the droplet motion and deformation are also studied and compared. This characterization will help the optimization of the electro-driven deformation and motion of sessile droplets for a variety of engineering and technological applications.

## 2. Construction of Dynamic Model

### 2.1. Model Description

**Figure 1:**
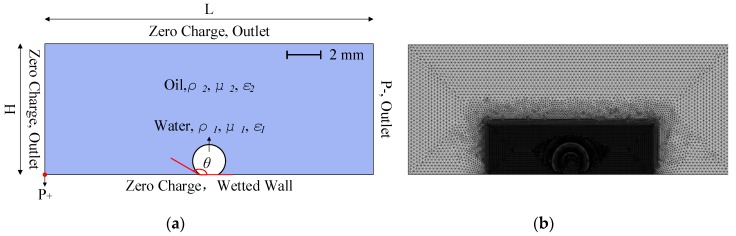
(**a**) Computational domain and boundary conditions with (**b**) the example of mesh used in simulations.

The two-dimensional simulation model and corresponding boundary conditions based on previous experimental conditions and theoretical research [13,14,15,16] is developed as shown in Figure 1a. The computation area contains the droplet and oil phase. A droplet with a certain volume is placed in the middle of substrate surfaces and forms a certain contact angle *θ*. The substrate bottom surface is considered to be in a wetted wall condition, the top and side surfaces of the computational domain are considered to be pressure outlet boundaries to ensure there are no pressure and velocity gradient effects. In order to simplify the problem and highlight the effect of a non-uniform electric field, the electric field structure is simplified to a pin-plate electrode structure that locates at the left and right sides, respectively. Specific model sizes and parameter values used in these simulations are listed in Table 1.

The model is divided by free triangle meshes and there is the refined meshes in and near the droplet region. By using virtual operations and mesh controlled edges, a rectangular virtual domain with higher resolution than the remaining domain is introduced to decrease the number of elements and thus reduces the computational load considerably. An example of such a mesh is shown in Figure 1b and a mesh independence study is performed to validate the numerical stability of the simulation. The conditions of V_+_ = 15 kV, Z = 4 mm^3^ and *θ* =150° are used as a mesh correction benchmark. By changing the number of meshes from 46,646 (mesh 1) to 60,996 (mesh 2), the final horizontal displacement of the droplet with 3 s is less than 2.77%. The error caused by finite mesh is relatively small and the mesh 1 is sufficiently precise for the present model. Therefore, the structure of the mesh 1 above is adopted for all cases in this study.

### 2.2. Two-Phase Flow Equations 

The whole system can be considered as an isothermal and incompressible laminar flow due to small changes in temperature and velocity. Based on mass and momentum conservation, the governing equations for both phases are the incompressible Navier–Stokes equations:

(1) $$\nabla \cdot \vec{u}=0$$

(2) $$\rho \frac{\partial \vec{u}}{\partial t}+\rho \left(\vec{u}\cdot \nabla \right)\vec{u}=\nabla \cdot \left[-pI+\mu \left(\nabla \vec{u}+{\left(\nabla \vec{u}\right)}^{T}\right)\right]+\rho g+F_{st}+F_{e}$$

where $\vec{u}$ (m/s) is the velocity, *ρ* (kg/m^3^) is the density, *p* (Pa) is the pressure, *μ* (Pa·s) is the dynamic viscosity, I is the unit matrix, g (m^2^/s) is the acceleration of gravity, *F_st_* (N/m^3^) is the surface tension per unit volume on the water–oil interface, and *F_e_* (N/m^3^) is the electric stress per unit volume respectively. 

Phase field method, an effective tool for exploring the multiphase flow behaviors and interfacial phenomena, used in this paper is relatively effective in multiphase flow research efforts. The water–oil phase interface is described by a phase field variable, *φ*, whose evolution is governed by the Cahn–Hilliard equation [19]:

(3) $$\frac{\partial \phi }{\partial t}+\vec{u}\cdot \nabla \phi =\nabla \gamma \nabla G$$

where *γ* (m^3^·s/kg) is the mobility parameter that controls the diffusion scale, and (Pa) is the chemical potential at the phase interface respectively. The formulas of *γ* and *G* are depicted as follows:

(4) $$\gamma =\chi e_{pf}{}^{2}$$

(5) $$G=\lambda \left[-\nabla^{2}\phi +\frac{\phi \left(\phi^{2}-1\right)}{e_{pf}{}^{2}}\right]$$

where *χ* (m·s/kg) is the mobility tuning parameter and set to 1 m·s/kg that is a good starting point for current models, *e_pf_* (m) is capillary width that scales with the thickness of the interface and is set to R/20 (R is the droplet radius), and *λ* (N) is the mixing energy density respectively. *λ* and *e_pf_* are related to the surface tension coefficient, *α* (N/m), through the equation:

(6) $$\alpha =\frac{2\sqrt{2}}{3}\frac{\lambda }{e_{pf}}$$

The parameters appearing in equations are determined by the phases. These quantities are modeled as global variables that vary across the interface with respect to volume fraction. Therefore, the density and dynamic viscosity in the two-phase flow are defined as:

(7) $$\rho =\rho_{1}V_{f1}+\rho_{2}V_{f2}$$

(8) $$\mu =\mu_{1}V_{f1}+\mu_{2}V_{f2}$$

where subscript 1 and 2 represent the water phase and oil phase respectively. The volume fraction $V_{f}$ of phase 1 and phase 2 is calculated as:

(9) $$\{\begin{array}{c} V_{f1}=\frac{1-\phi }{2}\\ V_{f2}=\frac{1+\phi }{2} \end{array}$$

Phase 1 corresponds to the domain where *φ* = −1, and phase 2 corresponds to the domain where *φ* = 1. The interface between the two fluids is evolved by a fixed contour of a phase field function *φ* = 0.

The surface tension force *F_st_* is added to the Navier-Stokes equations as a body force by multiplying the chemical potential of the system by the gradient of the phase field variable.

(10) $$F_{st}=G\nabla \phi$$

### 2.3. Electric Field Equations

An idealized case that the both phases (the droplet and the oil) are leaky dielectrics in a DC electric field is considered. Therefore, the governing equation for the electric field is expressed by Maxwell’s equation [30]:

(11) $$-\nabla \cdot \left(\varepsilon_{0}\varepsilon \vec{E}\right)=0$$

where *ε*_0_ (F/m) is the free space permittivity, and *ε* is the relative permittivity, and $\vec{E}$ (V/m) is the electric field strength, which can be expressed as the gradient of electric potential, *V* (V):

(12) $$\vec{E}=-\nabla V$$

The relative permittivity *ε* can be calculated similarly to the density and viscosity:

(13) $$\varepsilon =\varepsilon_{1}V_{f1}+\varepsilon_{2}V_{f2}$$

The electric force *F_e_*, as a source term of the Navier-Stokes equations, can be calculated by the divergence of the Maxwell stress tensor, *τ^M^* (SI unit: N/m^2^) [31,32]. Under the conditions of the perfect dielectric model and incompressible fluid, the electric force per unit volume is given as:

(14) $$F_{e}=\nabla \cdot \tau^{M}=\nabla \varepsilon_{0}\varepsilon \left[\vec{E}\vec{E}-\frac{1}{2}E^{2}I\right]$$

The electric force is along the normal direction of two-phase interface. In the case of non-uniform electric fields, the electric force of the polarized dielectric is referred to as the DEP force. 

## 3. Results and Discussion

The motion and deformation of droplets on the substrate surface under electric fields are mainly affected by the inertial force, viscous force, electric field force, surface tension and substrate adhesion. The characteristic phenomena are determined by the relative importance of the forces in the flow. It is useful to calculate several dimensionless numbers based on the relative magnitudes of key physical parameters before performing full time-dependent simulations, which characterize fluid behavior in multiphase and microfluidic flows. In many cases it is not necessary to calculate dimensionless numbers accurately, and just simply estimating its magnitude is sufficient [33]. The two-phase characteristic flow under the electric field is mainly determined by the Reynolds number (*Re*), capillary number (*Ca*) [34,35] and electric Bond number (*Bo_E_*) [33]:

(15) $$Re=\frac{\rho_{1}uR}{\mu_{1}}$$

(16) $$Ca=\frac{\mu_{1}u}{\alpha }$$

(17) $$Bo_{E}=\frac{\varepsilon_{0}\varepsilon RE^{2}}{\alpha }$$

here the characteristic length of the two-phase flow is represented by the droplet radius, R (mm), which can be calculated at the given droplet volume and contact angle:

(18) $$R={\left(Z/(\frac{4\pi }{3}-\frac{\pi }{3}{\left(1+\cos\theta \right)}^{2}(3-(1+\cos\theta )))\right)}^{\frac{1}{3}}$$

The Re number, *Ca* number and *Bo_E_* number are, respectively, the ratio of inertial forces to viscous forces, viscous forces to surface tension forces and electric forces to surface tension forces. Using these definitions, the Re number varies between about 0.9 and 1.5, which means viscous forces are sufficiently strong to prevent the flow from becoming turbulent; the *Ca* number ranges between about 0.014 and 0.024, which means the interface shape and velocity distribution are driven by the surface tension; the *Bo_E_* number varies between about 3.3 and 5.6, which means electric field force can overcome the surface tension and change the interface shape and velocity distribution. Therefore, the above assumptions and equations are applicable to the numerical simulation study of this paper.

In this paper, COMSOL Multiphysics 5.4 (COMSOL, Inc., Burlington, MA, USA), the multiphysical field coupling software, is employed to perform the numerical simulations. The second-order quadratic basic functions used for the phase-field varies, the velocity is filed, and the electric potential is recorded, while the pressure is solved using linear basic functions. The transient solver PARDISO is adopted with phase initialization. The time step is set to 0.01 s and 0.02 s according to the degree of the droplet movements. The time step 0.01 s is used to calculate the droplet movement within 0 to 0.2 s, and the time step 0.01 s is used to calculate the remaining time period. The fully coupled system is solved by the backwards Euler method.

### 3.1. Deformation and Motion of the Droplet

**Figure 2:**
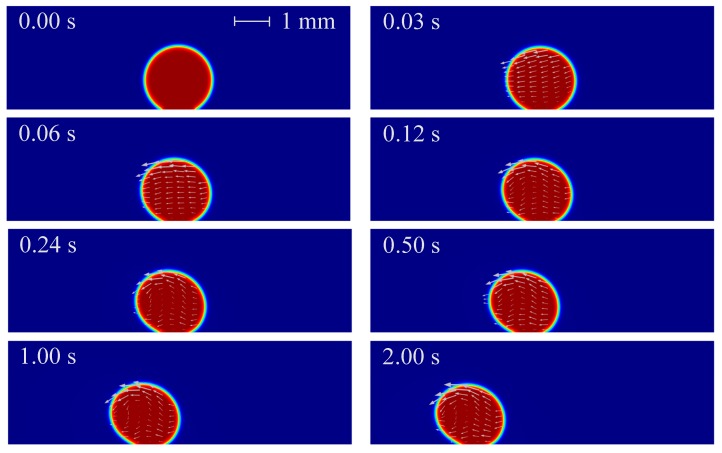
Time-lapse evolutions of droplet morphology (V_+_ = 15 kV, Z = 4 mm^3^ and *θ* = 150°).

The droplet with the condition of V_+_ = 15 kV, Z = 4 mm^3^ and *θ* = 150° is considered first, whose angle is the demarcation contact angle between hydrophobic and superhydrophobic structures. The time-lapse evolutions and local velocity field inside the droplet are shown in Figure 2. As can be seen that under the effect of non-uniform electric fields, the droplet deforms to the left offset and slides to the left side along the substrate. During the motion, the droplet offset is becoming larger, corresponding to this is the gradually increasing advancing angle and the gradually deceasing receding angle. The droplet finally moves to the left side, which is the direction of increasing electric field strength.

The above phenomenon can be explained by the non-uniform DEP force of droplets under non-uniform electric fields [36]. In the absence of electric fields, the stationary sessile droplet on substrate surfaces maintains its spherical cap shape and forms a certain contact angle because of the effect of interfacial tension. The droplet is polarized under the effect of electric fields: the electric dipole moments are generated along the direction of the electric field inside the droplet, and polarization charges are generated on both sides of droplet surface. As the electric field strength increases, the charge density on both sides of the droplet increases, which means the electric field force on both sides of the droplet also increases. When electric fields are non-uniform, the electric dipole is subjected to an uneven electric field force, so that the force on both sides of the droplet is uneven. When the electric field force on both sides of the droplet is large enough, the droplet can be deformed against the surface tension, and even moves against the substrate adhesion. When the polarization degree of the droplet is higher than that of the surrounding phase, the positive DEP force occurs, resulting in a net DEP force pointing to the direction of increasing electric field strength; by contrast, when the polarization degree of the droplet is lower than that of the surrounding phase, the negative DEP force occurs, resulting in a net DEP force pointing to the direction of decreasing electric field strength. In this paper, the relative permittivity of the droplet phase (*ε*_1_ = 80) is much larger than that of the oil phase (*ε*_2_ = 2.2), so that there is a positive DEP force acting on the droplet under non-uniform electric fields. In addition, there is an adhesion force on the contact surface between the droplet and the substrate, resulting in sluggish motions of the bottom surface of the droplet, thus causing droplet offset. In summary, under the combined action of the gravity, surface tension, non-uniform DEP force and substrate adhesion, the droplet deforms and moves to the left side, which is the direction of increasing electric field strength.

**Figure 3:**
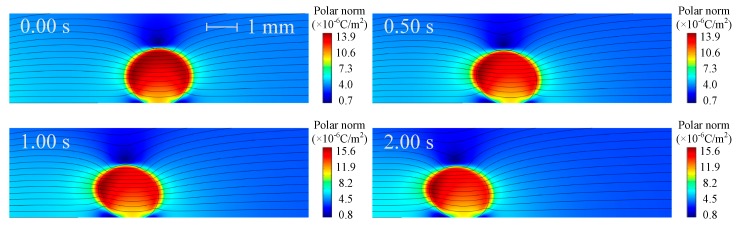
Electric polarization and electric field lines around the droplet (V_+_ = 15 kV, Z = 4 mm^3^ and *θ* = 150°).

**Figure 4:**
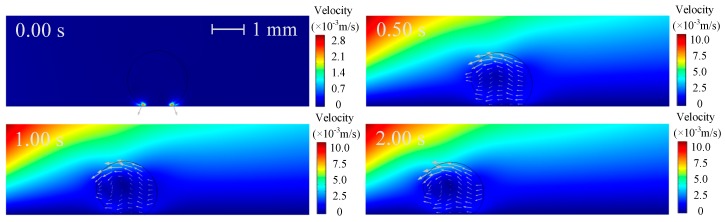
Velocity distribution around the droplet (V_+_ = 15 kV, Z = 4 mm^3^ and *θ* = 150°).

**Figure 5:**
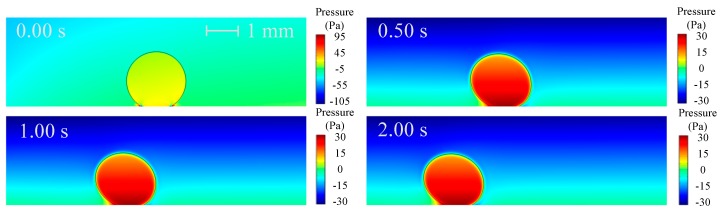
Pressure distribution around the droplet (V_+_ = 15 kV, Z = 4 mm^3^ and *θ* = 150°).

The electric polarization degree of the droplet, represented by the norm of polarization charge, is shown in Figure 3, in which the black line is electric field lines around the droplet. As can be seen that the electric polarization degree and electric lines vary with the droplet motion and deformation pattern. There is a greater polarization degree inside the droplet and electric field lines shrink in the droplet phase. More specifically, the polarization degree of the droplet increases with time, and the polarization degree on both sides of the droplet is different: the left is higher and the right is lower. The reason for these phenomena is due to the fact that the droplet is more easily polarized than the oil (*ε*_1_ > *ε*_2_) and the polarization degree increases with the increase of electric field strength. As time increases, the droplets is closer to the pin electrode with higher electric field strength, so that the polarization degree inside the droplet increases and the difference in the polarization degree on both sides of the droplet is more distinct. The velocity and pressure distributions are shown in Figure 4 and Figure 5, respectively.

**Figure 6:**
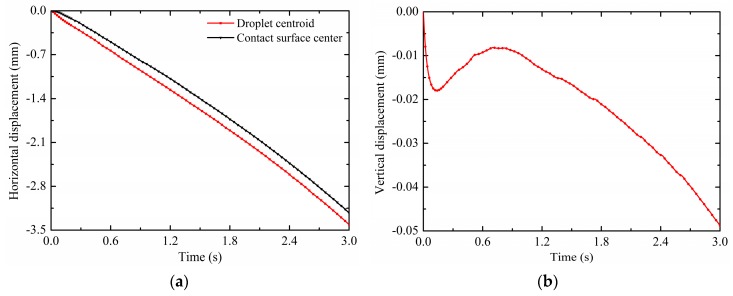
Variations in (**a**) the horizontal displacement and (**b**) vertical displacement (V_+_ = 15 kV, Z = 4 mm^3^ and *θ* = 150°).

In order to quantitatively show the effects of electric fields on the droplet motion, variations of the droplet horizontal displacement *d_x_* (mm) and vertical displacement *d_y_* (mm) of droplet centroids are shown in Figure 6a,b. The droplet centroid is the value of the two-dimensional coordinates x and y of the droplet, which are calculated as $\iint \left(x\times V_{f1}\right)/\iint V_{f1}$ and $\iint \left(y\times V_{f1}\right)/\iint V_{f1}$, respectively. It can be seen from Figure 6a that the droplet horizontal displacement value increases with time and is nearly linear. The negative sign represents the horizontal displacement direction of the droplet from the right to the left. Within 3 s, the horizontal displacement value of droplet centroids reaches 3.4106 mm. Comparing the horizontal displacement curves of droplet centroids and contact surface centers, the former responds to the DEP force relatively quickly about 0.02 s. This means that under the effects of non-uniform electric fields, the droplet first deforms against the surface tension, that is, the droplet centroid begins to move first; then the droplet is driven to move against the substrate adhesion, this is, the droplet-substrate contact surface center moves later. In addition, there is always a difference between the droplet centroid horizontal displacement and the contact surface horizontal displacement, indicating that the droplet is deformed. It can be seen from Figure 6b that the vertical displacement of the droplet centroid firstly moves downward quickly under the non-uniform DEP force, and then reverts upward due to the release of the surface tension caused by the droplet deformation. The maximum revert displacement of the droplet is 0.0082 mm at 0.7 s. The negative sign represents the vertical displacement direction of the droplet from the up to the down. This is because the pin electrode is located below the droplet, so that the vertical component of the non-uniform DEP force makes the droplet move vertically downward. After that, the vertical displacement of the droplet centroid gradually moves downward due to the enhancement of the electric field strength as the droplet gets closer to the pin electrode.

**Figure 7:**
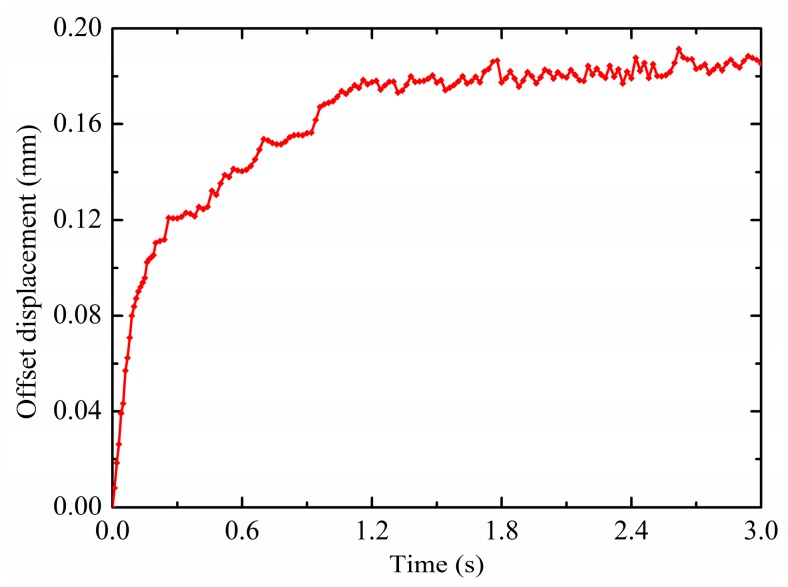
Variations in the offset displacement (V_+_ = 15 kV, Z = 4 mm^3^ and *θ* = 150°).

In order to show the effects of electric fields on the droplet deformation, the droplet offset displacement *d_o_* (mm) is defined as the horizontal displacement difference between the contact surface center and the droplet centroid, $d_{o}=d_{\mathrm{center}}-d_{x}$. The variation of the droplet offset displacement is shown in Figure 7. As displayed, the droplet offset displacement is sharply increased within 0.3 s. This is due to the fact that the droplet centroid moves faster in response to the non-uniform DEP force relative to the contact surface center. Then the increase in the droplet offset displacement becomes slow because the motive of the contact surface center is gradually synchronized with the droplet centroid. Finally the droplet offset displacement continues to increase almost steadily and keeps oscillating around 0.0091 mm. In the process of a non-uniform electric field driving droplet motion, the droplet does not maintain a constant deformation state, but the deformation relaxation occurs with a slight oscillation phenomenon due to its own inertia. By contrast with the one-time revival phenomenon of the vertical displacement, the oscillation phenomenon of the horizontal offset displacement exists all the time. In summary, the non-uniform electric fields can induce positive electrophoresis motion and deformation of the droplet. 

### 3.2. Effects of the Potential

**Figure 8:**
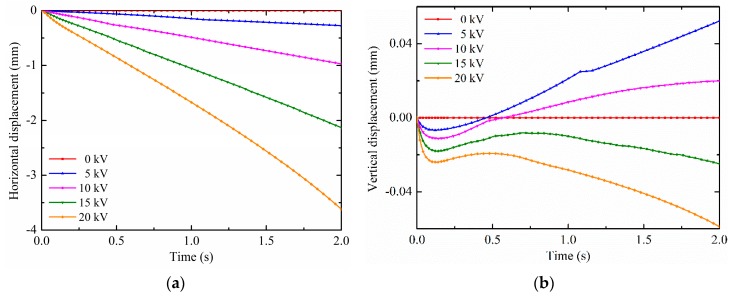
Effects of the potential on (**a**) the horizontal displacement and (**b**) vertical displacement.

**Figure 9:**
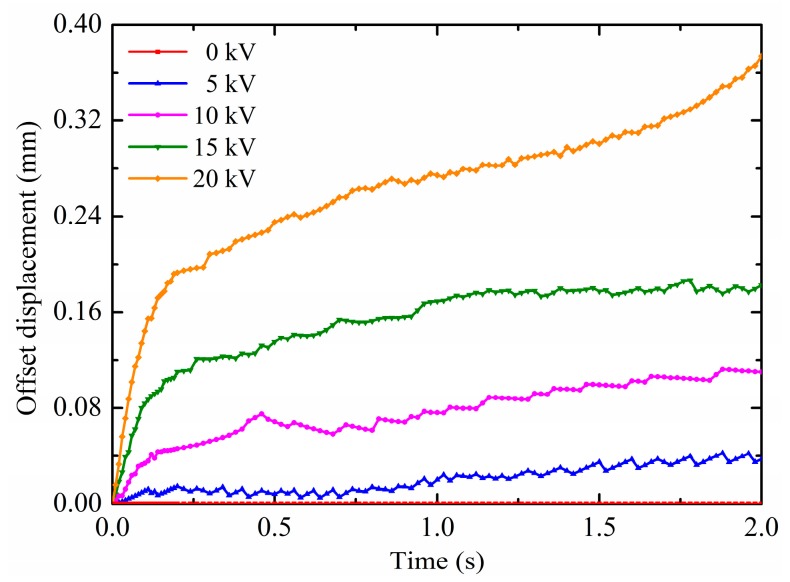
Effects of the potential on the offset displacement.

It has been revealed that the electrohydrodynamic phenomenon of the droplet with the contact angle of 150° under non-uniform electric fields, which the non-uniform polarization force can cause droplets to offset deform and move along substrate surfaces. Therefore, the electric field potential has an important impact on the droplet electro-driven deformation and motion. In this section, the effects of different pin electrode potentials on the deformation and motion are discussed. The droplet volume is 4 mm^3^ and the contact angle is maintained at 150°. There is the same droplet radius of 0.9890 mm which is calculated according to Equation (18). The results on the droplet motion of different pin potentials are shown in Figure 8a,b. It is observed from Figure 8a that the horizontal displacement is increased when increasing the potential. This is because the electric field intensity and its gradient increase with the increase in the pin electrode potential at the same pin-plate electrode distance. According to Equation (12) and Equation (14), the non-uniform DEP force on the droplet also increases. Therefore, the horizontal displacement is increased with increasing the potential. Moreover, as shown in Figure 8b, there are some differences in the patterns of the droplet vertical displacement for different potentials. The vertical displacement of the first revert of the droplet increases as the potential increases. For the low potential, the droplet vertical motion direction can change. For example, the droplet vertical displacement changes from negative to positive at about 0.45 s for the potential of 5 kV, and at about 0.57 s for the potential of 10 kV. But for the potentials of 15 kV and 20 kV, the droplet vertical displacement are always negative. The smaller the potential, the lower the vertical component of the non-uniform DEP force, and the smaller the binding effect on the droplet. Therefore, for the low potential, the release of the surface tension caused by the droplet deformation not only makes the droplet revert, but also makes the droplet vertical displacement span from negative to positive. In this paper, the vertical displacement for the low potential does not change from positive to negative. In addition, the results on the droplet deformation of different pin potentials are shown in Figure 9. The horizontal component of the DEP force causes the horizontal displacement of the droplet. As can been seen, because the non-uniform DEP force increases with the increase in the pin electrode potential at the same pin-plate electrode distance, the offset displacement is increased with the increase in the potential. The closer the droplet moves to the pin electrode, the greater the effect of the non-uniform DEP force, resulting in a rapid increase in droplet offset displacement, such as the droplet for the potential of 20 kV.

### 3.3. Effects of the Contact Angle

**Figure 10:**
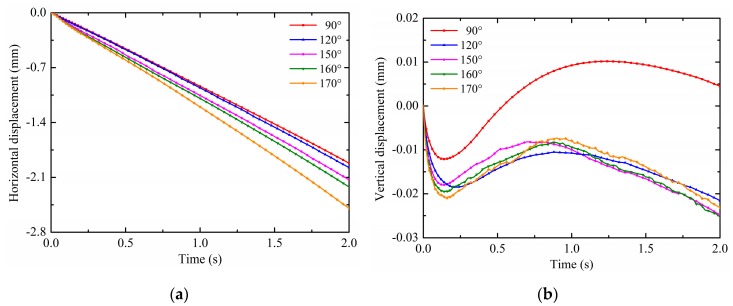
Effects of the contact angle on (**a**) the horizontal displacement and (**b**) vertical displacement.

**Figure 11:**
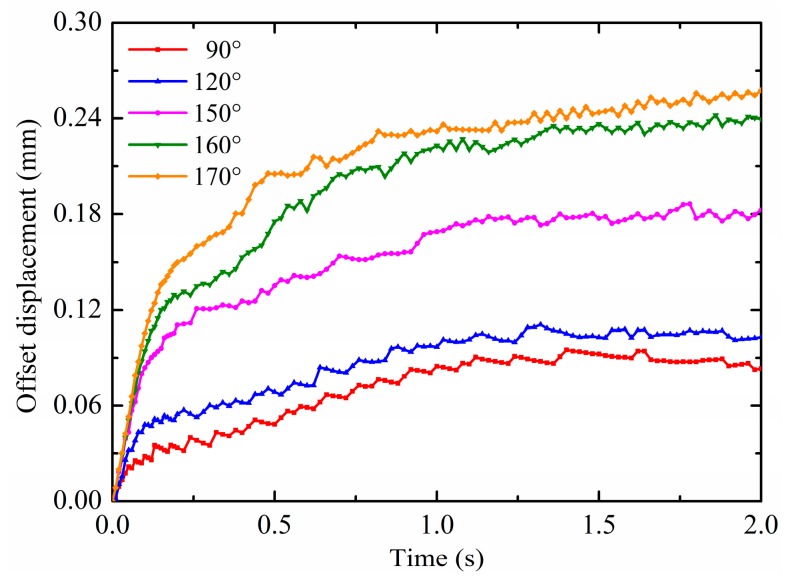
Effects of the contact angle on the offset displacement.

The contact angle is a measure of substrate wettability, that is, hydrophilic or hydrophobic. Typically, the contact angle less than 90° is referred to as hydrophilic; the contact angle greater than 90° is referred to as hydrophobic; and the contact angle greater than 150° is referred to as superhydrophobic. The contact angle is related to the surface tension and the substrate adhesion; thus, it can also affect the droplet deformation motion under non-uniform electric fields. Therefore, the effect of the contact angle is discussed in this section. The pin potential is set to 15 kV and the droplet volume is 4 mm^3^. According to Equation (18), the droplet radii are calculated as shown in Table 2. The results on the droplet motion of different contact angles are shown in Figure 10a,b. For ease of analysis, the calculated contact angles are divided into two categories: hydrophobic, which is 90° and 120°, and superhydrophobic, which is 150°, 160° and 170°. It is observed from Figure 10a that the horizontal displacement is increased by increasing the contact angle. The larger the contact angle, the more obvious the effect of non-uniform electric fields, as evidenced by the gap of the horizontal displacement curve. This is because the droplet with larger contact angles has the smaller contact area with the substrate surface, which means that the substrate adhesion is relatively small. Under the effect of same non-uniform electric fields, the droplet with larger contact angles are more susceptible to motion. Therefore, the horizontal displacement is increased with increasing the contact angle. Moreover, as shown in Figure 8b, there are great differences in the patterns of the droplet’s vertical displacement for different potentials. For the droplet with hydrophobic contact angles, 90° and 120°, the vertical displacement direction first changes at about 0.14 s and 0.21 s, respectively. The vertical displacement of the droplet with the contact angle of 90° changes from negative to positive after the revert. For the droplet with superhydrophobic contact angles, 150°, 160° and 170°, the vertical displacement direction first changes at about 0.13 s, 0.15 s and 0.18 s, respectively. The droplet vertical displacement is always negative. The smaller the contact angle, the lower the vertical component of the non-uniform DEP force, and the smaller the binding effect on the droplet. Therefore, the vertical displacement of the droplet with the contact angle of 90° changes from negative to positive after the revert. The stronger vertical component of the non-uniform DEP force results in the large revert vertical displacement and longer revert duration time, like *d_y_* (90°) < *d_y_* (120°), *d_y_* (150°) < *d_y_* (160°) < *d_y_* (170°). The reason why all contact angles cannot be compared is that there is an important factor affecting the droplet motion and deformation when the contact angle changes to cause a large change in the droplet radius, the additional pressure, Δp=2α/R. It is caused by the interfacial tension and toward the inside of the droplet. Among them, the radius of the droplet with contact angles of 90° and 120° varies greatly, so that the effect of the additional pressure is quite different from the other three. Furthermore, the results on the droplet deformation of different contact angles are shown in Figure 11. As can been seen, the same as the comparison of the horizontal displacement between contact angles, the offset displacement is increased with increasing the contact angle. As mentioned above, the droplets are more susceptible to motion and deformation as the contact angle increases under the effect of non-uniform electric fields.

### 3.4. Effects of the Droplet Volume

**Figure 12:**
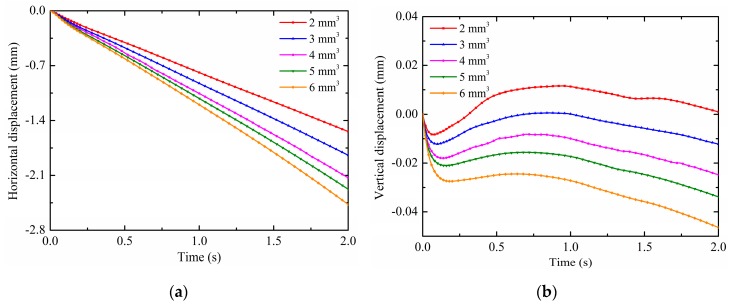
Effects of the droplet volume on (**a**) the horizontal displacement and (**b**) vertical displacement.

**Figure 13:**
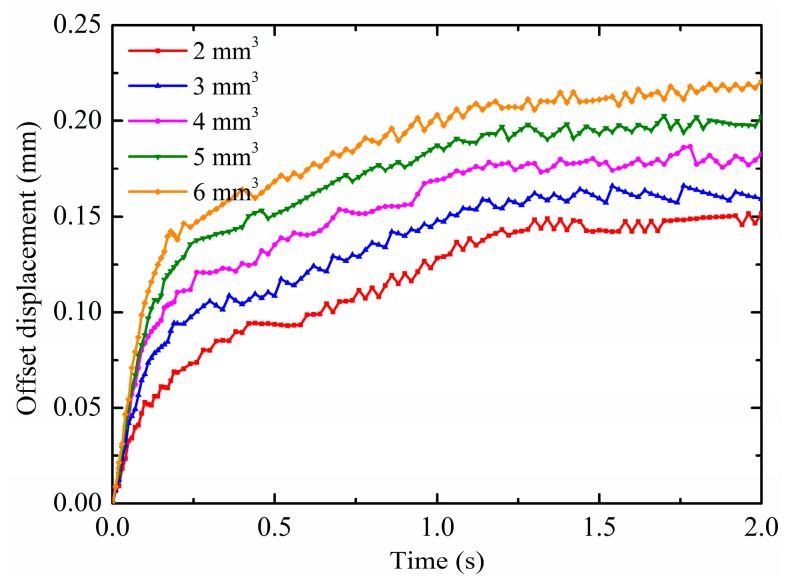
Effects of the droplet volume on the offset displacement.

The droplet volumes on substrate surfaces are not all the same in practice; therefore, they can also affect the droplet deformation and motion under non-uniform electric fields. For this reason, the effects of the droplet volume are discussed in this section. The pin potential is set to 15 kV and the contact angle is maintained at 150°. The droplet radius is calculated according to Equation (18) at different volumes as shown in Table 3. The results on the droplet motion of different droplet volumes are shown in Figure 12a,b. It is observed from Figure 12a that the horizontal displacement is increased as the droplet volume increases. The enhancement effect of non-uniform electric fields decreases as the droplet volume increases, as evidenced by the gap of the horizontal displacement curve. This is because the non-uniform DEP force is increased as the droplet volume increases, which has more polarization charges. On the other hand, the additional pressure decreases with increasing the droplet volume, which has larger droplet radii. At this time, the increase of the substrate adhesion due to the increase of the droplet volume can be ignored, so that the droplet with larger volume has larger horizontal displacement under the effect of the same electric fields. Moreover, as shown in Figure 12b, there are some differences in the patterns of the droplet vertical displacement for different droplet volumes. The vertical displacement of the first revert of the droplet increases as the droplet volume increases. For the small droplet volume, the droplet vertical displacement can change from negative to positive, such as the droplet with the volume of 2 mm^3^ and 3 mm^3^. For the droplet with the volume of 4 mm^3^, 5 mm^3^ and 6 mm^3^, the droplet vertical displacement are always negative. This is because the effect of gravity decreases as the droplet volume decreases, the release of the surface tension caused by the droplet deformation is sufficient to cause the droplet vertical displacement to change from negative to positive. Furthermore, the results on the droplet deformation of different droplet volumes are shown in Figure 13. As can been seen, the same as the comparison of the horizontal displacement between droplet volumes, the offset displacement is increased with increasing the droplet volume. As mentioned above, the droplets are more susceptible to motion and deformation as the contact angle increases under the effect of non-uniform electric fields. As mentioned above, the droplets are more susceptible to motion and deformation as the droplet volume increases under the effect of non-uniform electric fields.

## 4. Conclusions

In this study, the electrohydrodynamics phenomena of sessile droplets on hydrophobic surfaces under non-uniform electric fields are simulated using the phase field method. Dynamics behaviors of the electro-driven deformation and motion of water droplets in the oil phase are analyzed. The results show that under the effect of non-uniform DEP force, the droplet moves along the substrate surface to the direction of increasing electric field strength, and is accompanied with a certain deformation of the offset displacement. More specifically, the horizontal displacement of the droplet increases with time; and a revival phenomenon occurs in the direction of the vertical displacement; for droplet offset displacement, it first increases sharply and then slowly rises to a relatively stable value. The slight oscillation phenomenon in the horizontal offset of the droplet always occurs due to the deformation relaxation and its own inertia. In addition, the effect of pin electric potentials, surface contact angles and droplet volumes on the droplet motion and deformation are also studied and compared. The results show that the horizontal displacement and offset displacement of the droplet increase with the increase of pin potentials, contact angles and droplet volumes. For the vertical displacement, it is found that during the first revert process, the release of the surface tension caused by the droplet deformation can make the droplet with low potentials, small contact angles or small droplet volumes span from negative to positive. On the contrary, due to the effect of the non-uniform DEP force, the vertical displacement of the droplet with high potentials, and larger contact angles or large droplet volumes is always negative although it reverts. The results obtained in this study will be helpful for the future operation encountered in the electro-driven deformation and motion of droplets on hydrophilic and hydrophobic surfaces.

## Figures and Tables

**Table 1:** Simulation parameter setting.

Parameter	Symbol	Value	Units
Plate electrode potential	V_−_	0	kV
Pin electrode potential	V_+_	0–20	kV
Droplet volume	Z	2–6	mm^3^
Droplet contact angle	*θ*	90–170	°
Length of air domain	L	20	mm
Height of air domain	H	8	mm
Permittivity of vacuum	*ε*_0_	8.85 × 10^−12^	F/m
Acceleration of gravity	g	9.81	m^2^/s
Density of water	*ρ*_1_	998	kg/m^3^
Dynamic viscosity of water	*μ*_1_	0.001	Pa·s
Relative permittivity of water	*ε*_1_	80	-
Conductivity of water	*σ*_1_	1 × 10^−5^	S/m
Density of oil	*ρ*_2_	884	kg/m^3^
Dynamic viscosity of oil	*μ*_2_	0.474	Pa·s
Relative permittivity of oil	*ε*_2_	2.2	-
Conductivity of oil	*σ*_2_	1 × 10^−4^	S/m
Surface tension	α	0.03	N/m

**Table 2:** Relations between droplet contact angles and radius at the same droplet of 4 mm^3^.

*θ* (°)	90	120	150	160	170
R (mm)	1.2407	1.0421	0.9890	0.9856	0.9848

**Table 3:** Relations between droplet volumes and radius at the same contact angle of 150°.

Z (mm^3^)	2	3	4	5	6
R (mm)	0.7850	0.8986	0.9890	1.0654	1.1321

## References

[1] Chen L., Wang X., Yang T., Ping H., Bennett P., Zheng Z., Yang Q.B., Perrie W., Edwardson S.P., Dearden G. (2018). Superhydrophobic micro-nano structures on silicone rubber by nanosecond laser processing. J. Phys. D Appl. Phys..

[2] Li J., Wei Y., Huang Z., Wang F., Yan X., Wu Z. (2017). Electrohydrodynamic behavior of water droplets on a horizontal super hydrophobic surface and its self-cleaning application. Appl. Surf. Sci..

[3] Fürstner R., Barthlott W., Neinhuis C., Walzel P. (2005). Wetting and self-cleaning properties of artificial superhydrophobic surfaces. Langmuir.

[4] Wang N., Xiong D.S., Li M.T., Deng Y.L., Shi Y., Yang K. (2015). Superhydrophobic surface on steel substrate and its anti-icing property in condensing conditions. Appl. Surf. Sci..

[5] Miljkovic N., Enright R., Nam Y., Lopez K., Dou N., Sack J. (2013). Jumping-droplet-enhanced condensation on scalable superhydrophobic nanostructured surfaces. Nano Lett..

[6] Ölçeroğlu E., Hsieh C.Y., Rahman M.M., Lau K.K.S., Carthy M.M. (2014). Full-field dynamic characterization of superhydrophobic condensation on biotemplated nanostructured surfaces. Langmuir.

[7] Zeng J., Korsmeyer T. (2004). Principles of droplet electrohydrodynamics for lab-on-a-chip. Lab. Chip.

[8] Boreyko J.B., Zhao Y., Chen C.H. (2011). Planar jumping-drop thermal diodes. Appl. Phys. Lett..

[9] Tian L., Gao M., Gui L. (2017). A Microfluidic Chip for Liquid Metal Droplet Generation and Sorting. Micromachines.

[10] Mugele F., Baret J.C. (2005). Electrowetting: From basics to applications. J. Phys. Condens. Matter..

[11] Nam Y., Kim H., Shin S. (2013). Energy and hydrodynamic analyses of coalescence-induced jumping droplets. Appl. Phys. Lett..

[12] Liu X., Cheng P., Quan X. (2014). Lattice Boltzmann simulations for self-propelled jumping of droplets after coalescence on a superhydrophobic surface. Int. J. Heat Mass Tran..

[13] Takeda K., Nakajima A., Murata Y., Hashimoto K., Watanabe T. (2002). Control of water droplets on superhydrophobic surfaces by static electric field. Jpn. J. Appl. Phys..

[14] Sakai M., Kono H., Nakajima A., Sakai H., Abe M., Fujishima A. (2010). Water droplets’ internal fluidity during horizontal motion on a superhydrophobic surface with an external electric field. Langmuir.

[15] Zhu Y., Haji K., Otsubo M., Honda C., Hayashi N. (2016). Electrohydrodynamic behaviour of water droplet on an electrically stressed hydrophobic surface. J. Phys. D Appl. Phys..

[16] Wei Y., Li J., Huang Z., Yan X. Electrical driven rolling behavior of water droplet on a super hydrophobic surface. Proceedings of the ICHVE International Conference on High Voltage Engineering and Application.

[17] Adamiak K. (2006). Capillary and electrostatic limitations to the contact angle in electrowetting-on-dielectric. Microfluid. Nanofluidics.

[18] Bacri J.C., Salin D., Massart R. (1982). Study of the deformation of ferrofluid droplets in a magnetic field. J. Phys. Lett..

[19] Adzima B.J., Velankar S.S. (2006). Pressure drops for droplet flows in microfluidic channels. J. Micromech. Microeng..

[20] Moghtadernejad S., Tembely M., Jadidi M., Esmail N., Dolatabadi A. (2015). Shear driven droplet shedding and coalescence on a superhydrophobic surface. Phys. Fluids.

[21] Moghtadernejad S., Mohammadi M., Jadidi M., Tembely M., Dolatabadi A. (2013). Shear Driven Droplet Shedding on Surfaces with Various Wettabilities. SAE Int. J. Aerosp..

[22] Barwari B., Burgmann S., Janoske U. (2019). Hydrodynamic instabilities of adhering droplets due to a shear flow in a rectangular channel. Chem. Ing. Tech..

[23] Karsch S., Düsterer S., Schwoerer H., Ewald F., Habs D., Hegelich M. (2003). High-intensity laser induced ion acceleration from heavy-water droplets. Phys. Rev. Lett..

[24] Rowland S.M., Lin F.C. (2006). Stability of alternating current discharges between water drops on insulation surfaces. J. Phys. D Appl. Phys..

[25] Nudurupati S., Janjua M., Aubry N., Singh P. (2008). Concentrating particles on drop surfaces using external electric fields. Electrophoresis.

[26] Liu Y., Kong X., Wu Y., Du B. (2019). Dynamic behavior of droplets and flashover characteristics for CFD and experimental analysis on SiR composites. IEEE Access.

[27] Nakajima A. (2011). Design of hydrophobic surfaces for liquid droplet control. NPG Asia Mater..

[28] Li J., Wei Y., Huang Z., Wang F., Yan X.Z. (2016). Investigation of the electric field driven self-propelled motion of water droplets on a superhydrophobic surface. IEEE Trans. Dielect. Electr. Insul..

[29] Bansal L., Sanyal A., Kabi P., Pathak B., Basu S. (2018). Engineering interfacial processes at mini-micro-nano scales using sessile droplet architecture. Langmuir.

[30] Lin Y. (2013). Two-phase electro-hydrodynamic flow modeling by a conservative level set model. Electrophoresis.

[31] Barman J., Shao W., Tang B., Yuan D., Groenewold J., Zhou G.F. (2019). Wettability manipulation by interface-localized liquid dielectrophoresis: Fundamentals and applications. Micromachines.

[32] Zhou T., Yeh L.H., Li F.C., Mauroy B., Joo S.W. (2016). Deformability-based electrokinetic particle separation. Micromachines.

[33] Du W., Chaudhuri S. (2017). A multiphysics model for charged liquid droplet breakup in electric fields. Int. J. Multiph. Flow.

[34] Taylor G.I. (1932). The Viscosity of a fluid containing small drops of another fluid. Proc. R. Soc. Lond. Ser. A.

[35] Vlahovska P.M. (2019). Electrohydrodynamics of Drops and Vesicles. Annu. Rev. Fluid Mech..

[36] Ji X., Xu L., Zhou T., Shi L., Deng Y., Li J. (2018). Numerical investigation of DC dielectrophoretic deformable particle–particle interactions and assembly. Micromachines.

